# Supplementary material for: Midkine is a dual regulator of wound epidermis development and inflammation during the initiation of limb regeneration
Source: eLife. 2020 Jan 14;9:e50765. doi: 10.7554/eLife.50765 (PMC6959999; doi:10.7554/eLife.50765)
Supplement: Transparent reporting form [file elife-50765-transrepform.pdf]

eLIFE

Westbrook Centre, Milton Road
Cambridge CB4 1YG
UK

P 01223 855340
W elifesciences.org
T @elife

eLife's transparent reporting form

We encourage authors to provide detailed information *within their submission* to facilitate the interpretation and replication of experiments. Authors can upload supporting documentation to indicate the use of appropriate reporting guidelines for health-related research (see EQUATOR Network), life science research (see the BioSharing Information Resource), or the ARRIVE guidelines for reporting work involving animal research. Where applicable, authors should refer to any relevant reporting standards documents in this form.

If you have any questions, please consult our Journal Policies and/or contact us: editorial@elifesciences.org.

Sample-size estimation

- You should state whether an appropriate sample size was computed when the study was being designed
- You should state the statistical method of sample size computation and any required assumptions
- If no explicit power analysis was used, you should describe how you decided what sample (replicate) size (number) to use

Please outline where this information can be found within the submission (e.g., sections or figure legends), or explain why this information doesn't apply to your submission:

Power analysis was not performed for sample size estimation. Sample size (N) is stated in the legend of all figures. Each N in Figures 3-7 represents a regenerating limb from a different axolotl.

Replicates

- You should report how often each experiment was performed
- You should include a definition of biological versus technical replication
- The data obtained should be provided and sufficient information should be provided to indicate the number of independent biological and/or technical replicates
- If you encountered any outliers, you should describe how these were handled
- Criteria for exclusion/inclusion of data should be clearly stated
- High-throughput sequence data should be uploaded before submission, with a private link for reviewers provided (these are available from both GEO and ArrayExpress)

Please outline where this information can be found within the submission (e.g., sections or figure legends), or explain why this information doesn't apply to your submission:

eLife Sciences Publications, Ltd is a limited liability non-profit non-stock corporation incorporated in the State of Delaware, USA, with company number 5030732, and is registered in the UK with company number FC030576 and branch number BR015634 at the address Westbrook Centre, Milton Road Cambridge CB4 1YG, UK | March 2019

This information can be found in the figure legends and methods sections. All experiments in Figures 3-7 were performed in biological replicates of the stated N. For quantification of WE thickness, % EdU+ cells, % TUNEL+ cells in Figures 3-7, a total of 2-3 sections (technical replicates) around the radius/ulna-containing regions of each limb were quantified for variability and averaged to obtain the value for the biological replicate. No data was excluded.

For the initial bulk sequencing experiment in Figure 1, the experiment was performed in triplicate, with each N as a pool of 12 regenerating limbs in each condition. Pooling was necessary to obtain enough cells for RNA extraction in the dividing cell (4N) fraction. The DMSO/iMDK sequencing experiment was performed in biological triplicate.

The raw and processed sequencing data has been deposited on GEO:
Full skin flap dataset: GSE132317, reviewer token access: qhspiowgzbgdjmt
DMSO/iMDK dataset: GSE132325, reviewer token access: qdyxwakmxxajtan

Statistical reporting

- Statistical analysis methods should be described and justified
- Raw data should be presented in figures whenever informative to do so (typically when N per group is less than 10)
- For each experiment, you should identify the statistical tests used, exact values of N, definitions of center, methods of multiple test correction, and dispersion and precision measures (e.g., mean, median, SD, SEM, confidence intervals; and, for the major substantive results, a measure of effect size (e.g., Pearson's r, Cohen's d)
- Report exact p-values wherever possible alongside the summary statistics and 95% confidence intervals. These should be reported for all key questions and not only when the p-value is less than 0.05.

Please outline where this information can be found within the submission (e.g., sections or figure legends), or explain why this information doesn't apply to your submission:

Details on this information can be found in the legends of each figure and in the statistical analysis section within the methods. An unpaired student's t-test was used for statistical analysis, except for the rescue experiment in which a one way ANOVA analysis was performed. Graphs are mean ± standard deviation.

(For large datasets, or papers with a very large number of statistical tests, you may upload a single table file with tests, Ns, etc., with reference to sections in the manuscript.)

Group allocation

- Indicate how samples were allocated into experimental groups (in the case of clinical studies, please specify allocation to treatment method); if randomization was used, please also state if restricted randomization was applied
- Indicate if masking was used during group allocation, data collection and/or data analysis

Please outline where this information can be found within the submission (e.g., sections or figure legends), or explain why this information doesn't apply to your submission:

Samples were allocated by either treatment condition (DMSO or iMDK) or genotype (WT or mk^{null} mutant). For the initial sequencing experiment, samples were allocated based on whether they were normal regenerating or full skin flap sutured limbs. Images were blinded and randomized for all imaging-based analyses.

Additional data files ("source data")

- We encourage you to upload relevant additional data files, such as numerical data that are represented as a graph in a figure, or as a summary table
- Where provided, these should be in the most useful format, and they can be uploaded as "Source data" files linked to a main figure or table
- Include model definition files including the full list of parameters used
- Include code used for data analysis (e.g., R, MatLab)
- Avoid stating that data files are "available upon request"

Please indicate the figures or tables for which source data files have been provided:

eLIFE
Westbrook Centre, Milton Road
Cambridge CB4 1YG
UK
P 01223 855340
W elifesciences.org
T @elife

The source data has been provided for the IPA analysis of the Z-scores for pathway activation/inhibition predictions for full skin flap sutured vs. normal regenerating limb samples in Figure 1, the quantification of double in situ hybridization data in Figure 2, and the *mk* receptor TPM values and transcript IDs for the heatmap in Figure 8.
